# Seedless Cu Electroplating on Ru-W Thin Films for Metallisation of Advanced Interconnects

**DOI:** 10.3390/ijms23031891

**Published:** 2022-02-08

**Authors:** Rúben F. Santos, Bruno M. C. Oliveira, Liliane C. G. Savaris, Paulo J. Ferreira, Manuel F. Vieira

**Affiliations:** 1 Department of Metallurgical and Materials Engineering, University of Porto, 4200-465 Porto, Portugal; up200803851@edu.fe.up.pt (B.M.C.O.); up201601884@edu.fe.up.pt (L.C.G.S.); mvieira@fe.up.pt (M.F.V.); 2 LAETA/INEGI–Institute of Science and Innovation in Mechanical and Industrial Engineering, 4200-465 Porto, Portugal; 3 International Iberian Nanotechnology Laboratory, 4710-330 Braga, Portugal; paulo.ferreira@inl.int; 4 Materials Science and Engineering Program, University of Texas at Austin, Austin, TX 78712, USA; 5 Mechanical Engineering Department and IDMEC, IST, University of Lisbon, 1749-016 Lisbon, Portugal

**Keywords:** Cu, Ru, W, interconnect, seedless, electroplating, acidic, diffusion barrier

## Abstract

For decades, Ta/TaN has been the industry standard for a diffusion barrier against Cu in interconnect metallisation. The continuous miniaturisation of transistors and interconnects into the nanoscale are pushing conventional materials to their physical limits and creating the need to replace them. Binary metallic systems, such as Ru-W, have attracted considerable attention as possible replacements due to a combination of electrical and diffusion barrier properties and the capability of direct Cu electroplating. The process of Cu electrodeposition on Ru-W is of fundamental importance in order to create thin, continuous, and adherent films for advanced interconnect metallisation. This work investigates the effects of the current density and application method on the electro-crystallisation behaviour of Cu. The film structure, morphology, and chemical composition were assessed by digital microscopy, atomic force microscopy, scanning and transmission electron microscopies, energy-dispersive X-ray spectroscopy, and X-ray diffraction. The results show that it was possible to form a thin Cu film on Ru-W with interfacial continuity for current densities higher than 5 mA·cm^−2^; however, the substrate regions around large Cu particles remained uncovered. Pulse-reverse current application appears to be more beneficial than direct current as it decreased the average Cu particle size.

## 1. Introduction

Increasing miniaturisation, well described by the famous Moore’s law, has made supercomputing-multitasking devices able to be downscaled for decades. Computers, smartphones, and smartwatches, are now considered fundamental in our modern lives, while their complexity and performance are increasing every year. For example, the recently launched A15 Bionic chip is capable of 15.8 billion operations per second, enabled by 15 thousand million transistors packed in a few square millimetres. As the chip performance increases, transistors and interconnects have been downsized to the nanometric scale, challenging the usability limits of the materials and their processing [1], whose reliability strongly impacts the integrated circuit (IC) performance and power consumption [2].

The Ta/TaN bilayer has been the industry standard for the Cu interconnect metallisation in IC fabrication [3]. TaN functions as a diffusion barrier against Cu migration into the dielectric layers, whereas Ta grants adhesion between Cu and TaN. Cu displays a relatively high intrinsic diffusion coefficient in Si as given by Equation (1) [4]:

(1) $$D_{int}=3\times 10^{-4}\times e^{\frac{0.18}{k_{B}T}}\;{\mathrm{cm}}^{2}\cdot s^{-1}$$

with $k_{B}$ and T being the Boltzmann constant and the absolute temperature, respectively, and which, in the absence of an effective diffusion barrier, will generate deep trap states, shortcut paths in case of agglomeration, and potentially form copper silicides, modifying the electronic structure and yielding an extensive crystal lattice expansion [5,6]. The relatively high electrical resistivity of TaN [7] plays a significant role in the global interconnect resistivity as the interconnect linewidth shortens. 

Furthermore, it is not possible to properly fill the interconnects by electroplating Cu directly on Ta, which requires a physical-vapour deposited (PVD) Cu seed layer to be grown before metallisation. Advanced interconnects require liners that are effective diffusion barriers, display low resistivity, and allow direct (seedless) Cu electroplating. Ru has been receiving considerable attention to replace Ta/TaN, since Ru displays a much lower resistivity than TaN and even Ta [8], a superior adhesion to Cu [9,10], and is compatible with seedless Cu electroplating. 

Ru is chemically inert and stable, contrasting with its counterparts, such as Co, which is prone to dissolution during conventional acidic electroplating, requiring electrolyte modification to be used in seedless diffusion barrier systems [11,12]. However, similar to other candidates, Ru alone is not effective as a diffusion barrier [13], thus, driving the research on coupling Ru with other species to improve the barrier properties against Cu diffusion, including Ru-Co [14], Ru-Cr [15], Ru-Mn [16,17,18], Ru-N [19], Ru-P [20,21], Ru-Ta(-N) [16,22,23], and Ru-W(-N) [16,24,25] compositions. 

Bias temperature stress measurements by H. Wojcik et al. [16] showed that Ru_50_-W_50_ has an outstanding performance up to 600 °C; however, the best seedless Cu electroplating lies in the Ru-rich layers. Several studies were successful in electroplating Cu directly on Ru [26,27,28,29,30], but attempts on seedless Cu electroplating on Ru-W, particularly on Ru_50_-W_50_, are still scarce in the literature. Currently, the electro-crystallisation of Cu on Ru-W and its dependence on process variables, such as the current application mode and density, are not well known. 

The development of submicron-thick electroplated (EP) Cu films directly on diffusion barrier layers is of particular interest for advanced interconnect metallisation. Especially, understanding how Cu electro-crystallises on Ru-W is essential to evaluate its eligibility as a substitute of Ta/TaN. Such knowledge is also essential to develop strategies to tackle the challenges/issues emerged by electroplating Cu on a substrate that is more complex than the conventional Cu(seed)/Ta/TaN. Therefore, this work focuses on the formation and growth of EP-Cu on equimolar Ru-W using a conventional acidic electroplating electrolyte.

## 2. Results and Discussion

**Figure 1:**
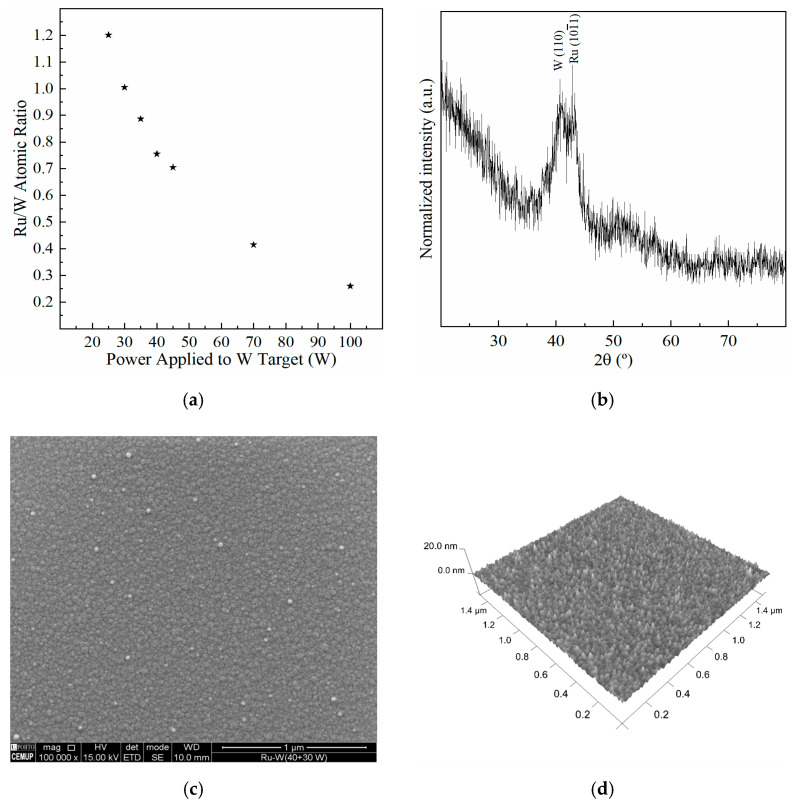
Ru/W atomic ratio vs. W target power, estimated by EDS (**a**). Sputtered Ru-W film X-ray diffractogram (**b**) and respective surface observed by SEM (**c**) and AFM (**d**).

The nucleation and growth of EP-Cu on Ru-W thin films are conditioned by the chemical composition of the substrate, namely the Ru/W ratio [25]. Different Ru-W thin film (≈20 nm thick) compositions were produced by varying the power applied to the W target between 25 and 100 W while maintaining the Ru target at 40 W. As the W target power decreases, the Ru/W ratio increases, reaching approximate atomic equimolarity at 30 W (Figure 1). The Ru/W atomic ratios were estimated by EDS, using a 15 keV electron beam and a collection time of 200 s to improve the signal-to-noise ratio, and using Ru L and W L emission lines for elemental quantification, which do not overlap with the Si or O peaks. 

Due to the large interaction volume created by the electron beam, X-rays are generated from within a few micrometers, which is well below the ≈20 nm thick Ru-W film. Accordingly, and due to the nature of the technique, the values shown in Figure 1a should be regarded as qualitative. The sputtering conditions and the thin nature of the deposit renders a film with a nanocrystalline/amorphous structure as suggested by XRD (Figure 1b), with a continuous and relatively smooth surface with an average roughness ($S_{a}$) of 0.7 nm (Figure 1c,d) determined using the *NanoScope* software 6.13R1 (Veeco Instruments Inc., Oyster Bay, NY, USA). 

Amorphisation is likely to be promoted by the addition of W. The reduction in the number of grain boundaries of a change towards an amorphous structure is a beneficial key aspect in the performance of diffusion barrier layers/films. A similar effect was observed with the addition of Cr in Ru-Cr films [15]. It is noteworthy that the flat substrate experimental approach herein used does not replicate the high aspect ratio shape characteristic of narrow interconnects; filling narrow vias/lines with Cu is a challenge itself. Nonetheless, due to its practical nature and simplicity of analysis, a flat substrate appeared to be a rather reasonable choice, given the objectives of this study.

Ru-W thin films with a near equimolar composition were selected and fabricated for the electrodeposition of Cu. Advanced interconnect metallisation requires direct Cu crystal nucleation and growth on diffusion barrier layers with complete substrate coverage and a submicrometric film that is uniform and compact. Thus, it is important to achieve conformal fill-up of narrow vias and shallow lines. In an ideal substrate with an infinite number of active sites for Cu nucleation, a uniform nuclei growth rate across its surface, and an electrodeposition efficiency equal to 1, the thickness, h, of the resulting film can be obtained through Faraday’s law and given by Equation (2):

(2) $$h=\frac{QM}{\rho eON}$$

where Q is the total transferred charge per unit area; M is the molecular mass of Cu; ρ is the density of Cu; e is the charge of the electron; O is the oxidation state for Cu ions; and N is Avogadro’s number. The values of Q can be determined with Equation (3), where J is the average current density and t is the electrodeposition time.

(3) Q=Jt

Different electroplating times, t, and average current densities, J, summarised in Table 1, were selected in order to comprise a total transferred charge of 300 mC, which produces a EP-Cu film of ≈110 nm thickness in the ideal substrate/reaction conditions mentioned above, using Equation (2). Both direct (DC) and pulse-reverse (PRC) currents were used in this study. In DC, a continuous cathodic current was applied, whereas, in PRC, the cathodic current was alternated with an anodic pulse. Table 1 summarises the electroplating conditions utilised, where $j_{c}$ and $j_{a}$ are the cathodic and anodic current densities, and $t_{c}$ and $t_{a}$ are the cathodic and anodic current times, respectively. In PRC, the values of J (conventionally assumed as cathodic) can be obtained from Equation (4).

(4) $$J_{PRC}=\frac{\left(j_{c}\times t_{c}\right)-\left(j_{a}\times t_{a}\right)}{t_{c}+t_{a}}$$

**Figure 2:**
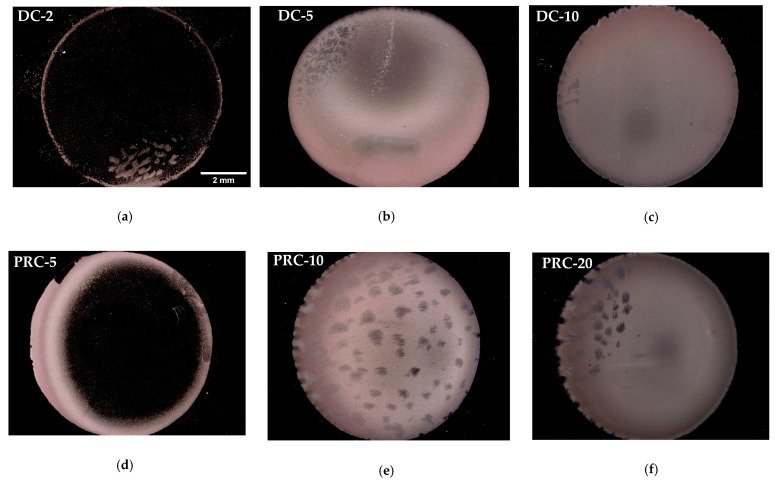
Digital microscopy images of the deposited Cu/Ru-W films with average current densities of 2 (**a**), 5 (**b**), 10 mA·cm^−2^ (**c**) in DC, and 3 (**d**), 6 (**e**), and 12 mA·cm^−2^ (**f**) in PRC.

The deposited Cu/Ru-W surface images (Figure 2) reveal that the average current densities of ≥5 mA·cm^−2^ produce a complete substrate coverage, whereas lower values of J result in a ring-like deposit. In complete coverage conditions, however, the EP-Cu film thickness and/or morphology are not uniform across the substrate’s surface, as is suggested by the differences in colour hue between its centre, which looks thinner, and its outer regions. The formation of hydrogen bubbles, due to the co-reduction of H^+^ ions into H^0^ and their coalescence into H_2_, is also apparent on the outer rim of the EP-Cu film, which intensifies with higher values of J. This effect is generally considered undesirable and dictates an electroplating efficiency to be under 1, implying that part of the transferred charge is consumed in reducing H^+^ ions.

**Figure 3:**
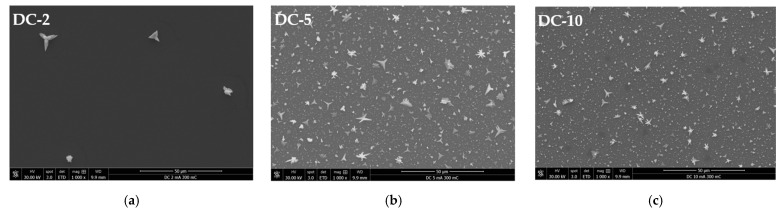

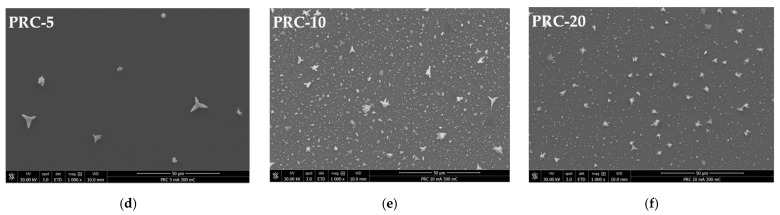
SEM images of the central/thinner regions of the deposited Cu/Ru-W films with average current densities of 2 (**a**), 5 (**b**), and 10 mA·cm^−2^ (**c**) in DC and 3 (**d**), 6 (**e**), and 12 mA·cm^−2^ (**f**) in PRC.

The SEM observation of the film surface over the central/thinner regions reveals a heterogeneous growth of Cu crystals (Figure 3). Average current densities of ≤3 mA·cm^−2^ result in a very low nucleation density. Under such conditions, growth is preferred over nucleation, since most of the transferred charge is used to growth the small number of nuclei that form. For J values of ≥5 mA·cm^−2^, many more Cu crystals with a variety of shapes and sizes nucleate and grow on the Ru-W substrate. It has been shown that, in Ru-Ta, the nucleation rate is inversely proportional to the particle size and increases with the current density [31]; however, it is interesting to note that, in this case, the relationship is not linear when considering the images in Figure 2. In fact, a rather small difference in J values between DC-5 and PRC-5 leads to a remarkable difference in substrate coverage.

**Figure 4:**
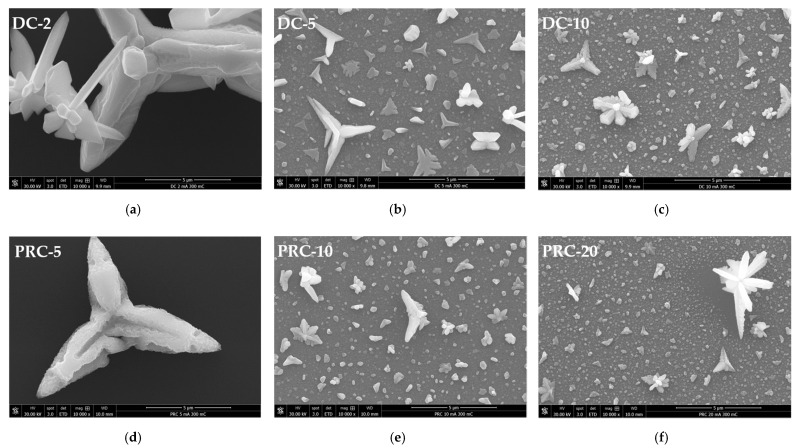
SEM images of the central/thinner regions of the as deposited Cu/Ru W films with average current densities of 2 (**a**), 5 (**b**), and 10 mA·cm^−2^ (**c**) in DC and 3 (**d**), 6 (**e**), and 12 mA·cm^−2^ (**f**) in PRC (same as in Figure 3 at 10-fold higher magnification).

A closer examination of the surface shows that, for average current densities of ≥5 mA·cm^−2^, the regions between large Cu crystals are covered with a relatively uniform and continuous Cu deposit (Figure 4). Under such electroplating conditions, a high density of nuclei form on the substrate in the very early stages of deposition, undergoing 3D growth thereafter. These nuclei grow at similar rates, arresting their 2D growth (i.e., parallel to substrate’s surface) when they impinge upon each other, forming relatively small-sized particles. 

This results in a uniform thickness deposit that covers most of the Ru-W film. However, some Cu nuclei experience particularly high growth rates with a preference for specific crystallographic directions [32], regardless of their orientation relative to the substrate’s surface. This translates into large Cu particles (>1 µm) with star-like/dendritic shapes dispersed along the surface. EP-Cu crystals with similar shapes were observed by U. Emekli and A. C. West [33] on glassy carbon and on 30 nm thick Ru films. The authors attributed such a morphology to particle densities lower than 10^8^ cm^−2^, when no other particle shape (and size) was present. 

This could explain the shape–size characteristics of the particles obtained at 2 (DC-2) and 3 mA·cm^−2^ (PRC-5) with particle densities in the order of 10^4^ cm^−2^ and average particle sizes, $\bar{d}$, of 6.53 and 5.05 µm (diameter), respectively, which were determined from SEM images. The large particles appeared to be in contact with the substrate over a small area, suggesting that such attachment can be easily disrupted during electroplating and/or specimen handling (i.e., rinsing, drying, and transporting), which could affect the particle density. For J values of 5 mA·cm^−2^ and higher, a wide range of particle sizes are present, and a correlation between size and shape is observed.

The star-like/dendritic shape is predominantly exhibited by larger (>1 µm) particles, whereas the smaller particles tend to display a more equiaxed shape, outnumbering the larger ones and impinging on each other, thus, covering most of the substrate. Due to this fact, it was not possible to determine the particle density in this range of J. Nevertheless, it is reasonable to infer that the particle density alone cannot explain the particle morphology. The particle growth rate appears to play an important role regarding particle shape, but its causes are unclear. Understanding the mechanisms underlying large Cu particle formation could be key to achieve more uniform EP-Cu films.

**Figure 5:**
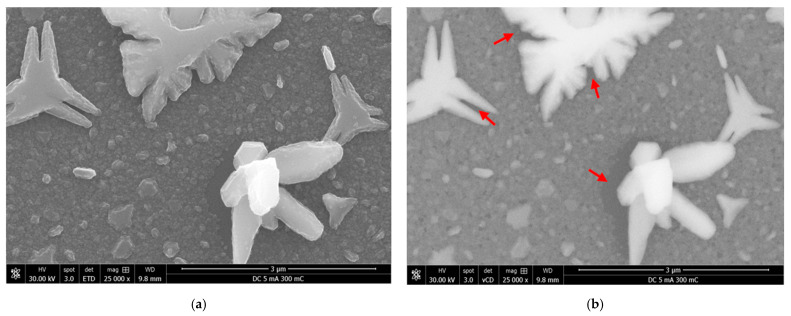
SEM images of deposited Cu/Ru-W films at 5 mA·cm^−2^ obtained by secondary (**a**) and backscattered (**b**) electrons.

Abnormal particle growth not only disrupts the film uniformity but also prevents nucleation and growth in their vicinity. The outcome is the presence of large Cu crystals surrounded by uncovered Ru-W substrate. This is seen in Figure 4f and is more evident in Figure 5. The fast-growth particles generate quite peculiar shapes, whereas the areas of the substrate around and underneath them are uncovered (Figure 5a). SEM images generated by backscattered electrons (in Figure 5b) highlight this effect by revealing darker regions in the vicinity of large Cu crystals (red arrows). 

**Figure 6:**
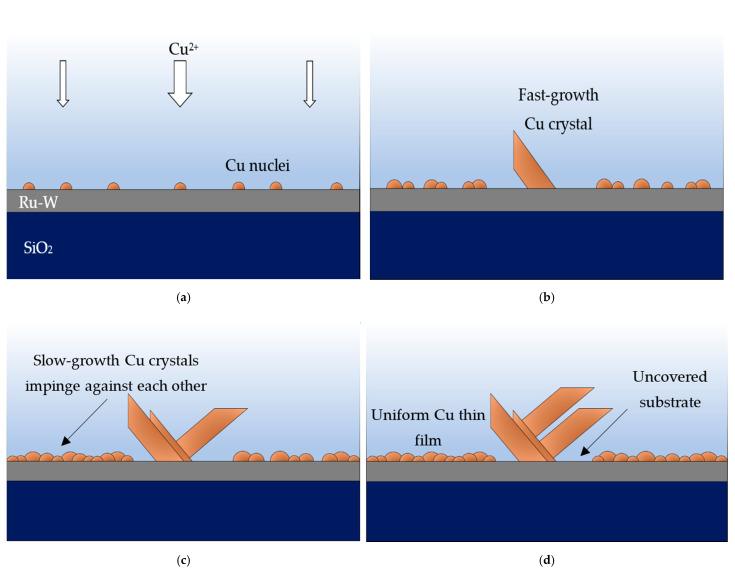
Early stages of Cu film formation on Ru-W. First nuclei appear (**a**) where a few undergo faster growth rate, consuming more Cu^2+^ (**b**). Slow-growing nuclei impinge on each other (**c**) and form a continuous Cu film, whereas fast-growing crystals hinder the substrate coverage underneath them (**d**).

In such cases, the absence of Cu produces a signal coming only from the substrate, which is predominantly (in volume) Si (a lighter element than Cu), which renders a darker contrast in the backscattered SEM image. The growth mechanism herein proposed is illustrated in Figure 6. 

**Figure 7:**
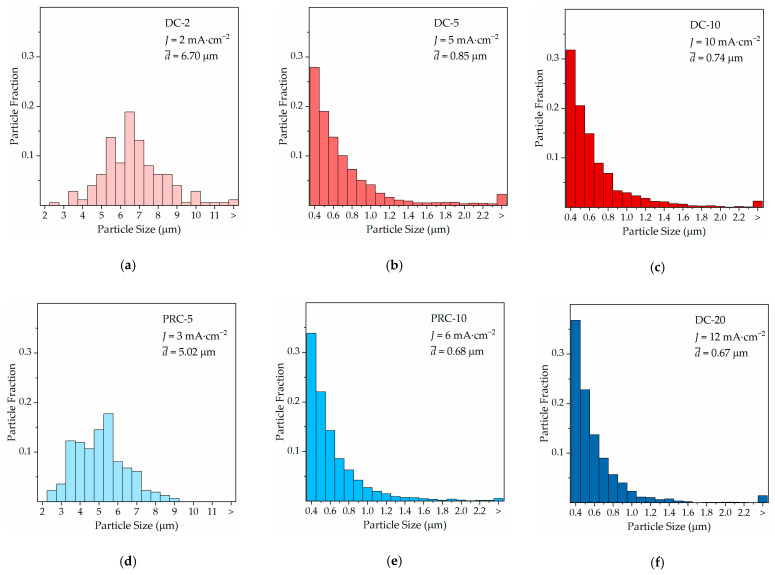
Particle size distributions for DC at J = 2 (**a**), 5 (**b**), and 10 mA·cm^−2^ (**c**) and for PRC at J = 3 (**d**), 6 (**e**), and 12 mA·cm^−2^ (**f**).

Increasing the current density from 5 to 10 mA·cm^−2^ in DC reduces the average Cu particle size. This can be regarded as beneficial since the uncovered regions of the substrate are seen around the large Cu particles. A comparison between DC-5 and PRC-10 is interesting because they have approximate values of J, 5 and 6 mA·cm^−2^, respectively, while the PRC-10 results in a smaller average particle size. In fact, PRC-10 results in a morphology finer than DC-10, even though J is 40% lower. This is clearly seen in the particle size measurements obtained by using the SEM micrographs to obtain particle size distributions (Figure 7).

Only particles with a minimum area of 8.48 × 10^−2^ µm^2^ (≈328 nm diameter) and not intersecting image edges were measured, across a minimum substrate area of 1.48 × 10^−2^ cm^2^ (10-times larger measurement areas were used for DC-2 and PRC-5) for each condition. PRC effectively reduces $\bar{d}$ by producing more particles <1 µm, an effect similar to increasing the current density in DC [34,35]. More than 50% of the measured abnormal particles are below 4.91 µm for DC-10, whereas, for PRC-10 and PRC-20, the median is 4.65 and 4.51 µm, respectively. 

Under such electroplating conditions, the cathodic process of ions reduction is interrupted by an anodic (reverse) pulse that allows preferential dissolution of the large, star-like/dendritic particles [36], resulting in a refined morphology. Hydrogen evolution in PRC-10 is seemingly close to that observed in DC-10, competing with Cu^2+^ reduction and decreasing the effective Cu deposition onto the Ru-W substrate.

**Figure 8:**
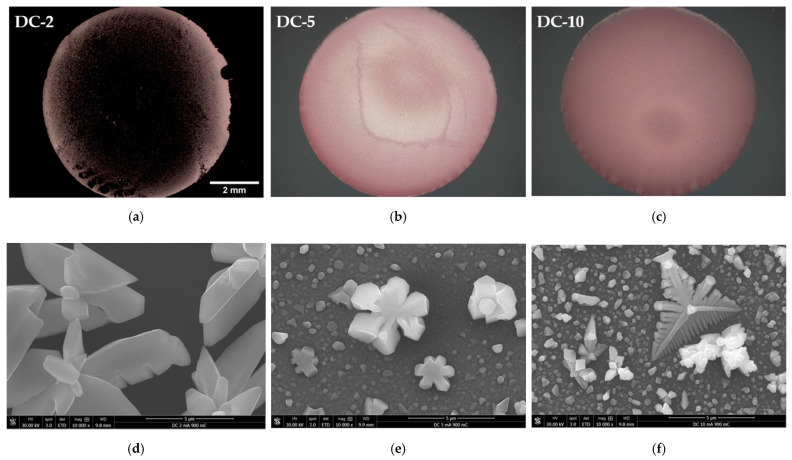
Digital microscope (**a**–**c**) and SEM images (**d**–**f**) of deposited Cu/Ru-W films at 2, 5, and 10 mA·cm^−2^, respectively, in DC with a total transferred charge of 900 mC.

**Figure 9:**
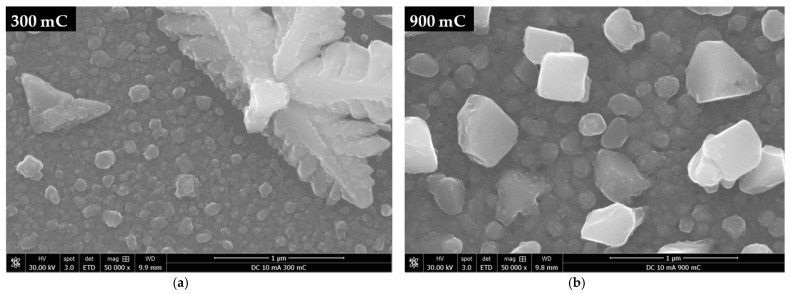
Cu films DC electroplated at 10 mA·cm^−2^ for 300 (**a**) and 900 mC (**b**) of transferred charge.

Longer deposition times were performed by applying a three-fold increase of the electroplating time, up to a total of 900 mC of transferred charge (Figure 8). At 2 mA·cm^−2^, no substantial substrate coverage is achieved (Figure 8a). The extra transferred charge is consumed to further grow the existing Cu particles, whereas the nucleation density does not significantly increase (Figure 8d). At J values of ≥5 mA·cm^−2^, the substrate coverage is consolidated, and the Cu film appears to be thicker with a more uniform colour tone (Figure 8b,c). Further growth increases the particle size (Figure 8e,f) and aids in reducing the coverage gaps around larger Cu particles (Figure 9).

**Figure 10:**
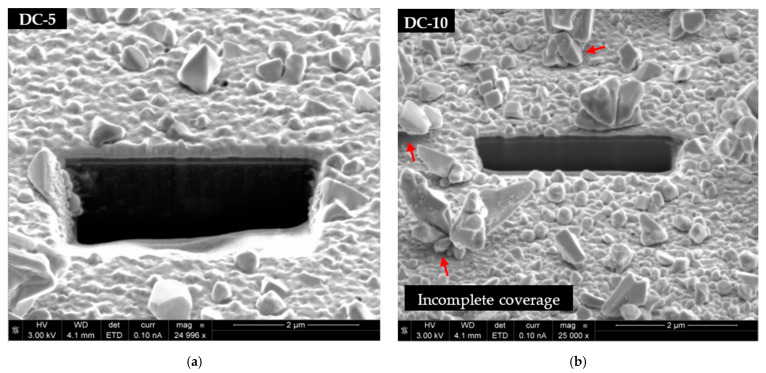
FIB-cut cross-sections of the Cu/Ru-W films deposited at 5 (**a**) and 10 mA·cm^−2^ (**b**) seen at a 52° tilt angle.

Some portions of the substrate underneath large particles remain uncovered as indicated by the red arrows (Figure 10). It is noteworthy that, in ideal electrodeposition conditions (i.e., uniform Cu film growth and electroplating efficiency equal to 1), these Cu films should be approximately 330 nm thick according to equation 1 and considering Q = 900 mC. Thicknesses measured from the FIB-cut cross-sections, however, obtain h values of ≈240–270 and ≈80–150 nm, for films electroplated at 5 and 10 mA·cm^−2^, respectively. The difference between the measured and calculated thicknesses can be implicitly attributed to the non-uniform distribution of the Cu mass across the substrate’s surface due to the faster growth of some Cu particles. 

**Figure 11:**
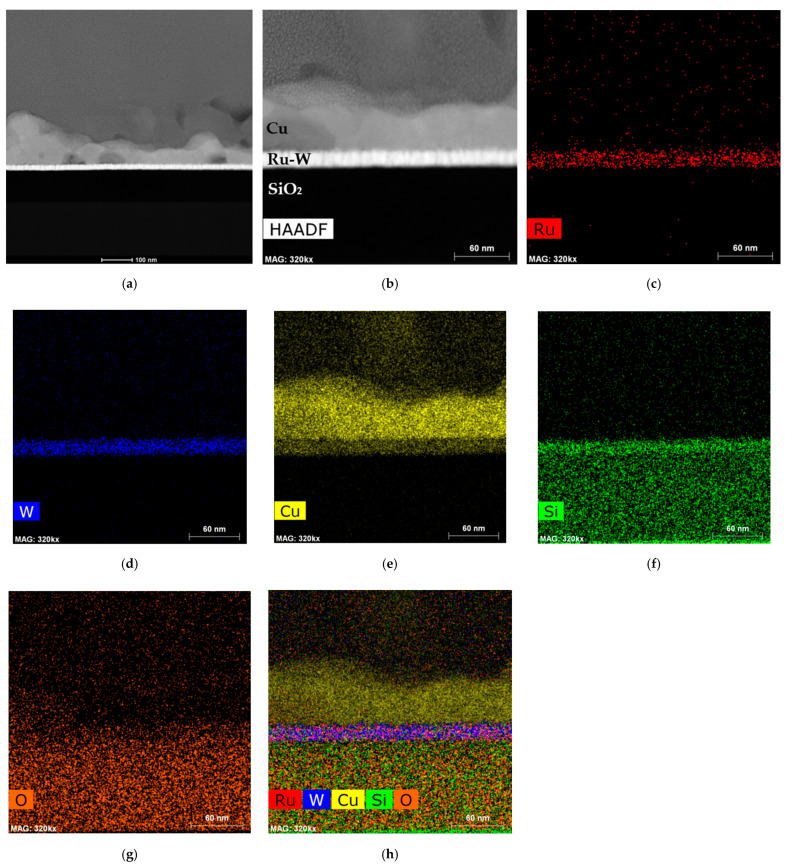
High-angle annular dark field (HAADF) image obtained in STEM mode of the DC-10 (900 mC) Cu/Ru-W interface (**a**). Higher magnification image (**b**) and respective elemental composition mapping generated by EDS are presented for Ru (**c**), W (**d**), Cu (**e**), Si (**f**), and O (**g**). Elemental EDS signal filtered and reconstructed by overlapping (**h**).

Thickness differences between DC-5 and DC-10 are supported by the difference in co-reduction of hydrogen, which consumes part of the transferred charge, which is more intense at 10 than at 5 mA·cm^−2^. Another interesting aspect to note is the seemingly continuous interface between Cu and Ru-W, which is essential to ensure interconnect performance and a requirement for proper adhesion. Figure 11 shows the cross-section of the DC-10 (900 mC) specimen observed by TEM. High-angle annular dark field (HAADF) imaging obtained in scanning transmission electron microscopy (STEM) mode reveals the stacked layers of EP-Cu/Ru-W/SiO_2_ with continuous interfaces between each layer (Figure 11a). 

The EP-Cu film thickness in this region ranges from 35 to 70 nm. The layer composition was determined by EDS elemental mapping. Ru and W are detected in the same layer (Figure 11b,c), Cu is present above (Figure 11d), and underneath the Ru-W barrier lies the SiO_2_ layer (Figure 11e,f). It is not reasonable nor plausible to assume that Cu atoms diffused into the Ru-W barrier layer from above, during or after electroplating, since the specimen handling and storing temperature was kept between 18 and 25 °C; the same could be said of Si atoms migrating from below. 

Instead, the relative proximity between Cu Kα (8.040 keV) and W Lα (8.396 keV) emission lines explain the signal coming from inside the barrier layer and attributed to Cu, by confusion with W. Similarly, the substantial Si signal coming from the barrier layer is also likely due to confusion with W, with the Si Kα (1.739 keV) emission line overlapping the intense W M (1.774 keV) peak. 

Considering this, a filtered and overlapped EDS signal for all elements is shown in Figure 11g, ascertaining the interfacial continuity and sound stacking of the layers. Clearly, improvement in EP-Cu morphology and a complete substrate coverage are required for Ru-W applicability as a seedless diffusion barrier layer. Stack electrical resistivity measurement and thermal stability assessment are additional fundamental steps in the process, which are currently under development.

## 3. Experimental

### 3.1. Ru-W Thin Film Deposition

A flat Ru-W/SiO_2_ bilayer was deposited on top of a p-type B-doped Si (100) wafer (Silicon Valley Microelectronics, Santa Clara, CA, USA) in two steps. In the first step, a ≈100 nm layer of SiO_2_ was grown by plasma-enhanced chemical vapour deposition (PECVD) with high radio frequency in a CVD MPX chamber (SPTS Technologies Ltd., Newport, UK). The SiO_2_/Si wafer was cut into small squares (15 mm × 15 mm) whereupon Ru-W films were deposited by DC magnetron sputtering in an ultra-high vacuum sputtering system (Kenosistec, Binasco, Italy). 

Ru and W were co-sputtered from their respective targets (99.95%, Testbourne Ltd., Basingstoke, UK) for 600 s. Films with different Ru/W ratios were obtained by applying a power bias between 25 and 100 W on the W target, while maintaining 40 W applied to the Ru target. The chamber Ar flux and working pressure were 20 sccm and 6.9 × 10^−1^ Pa, respectively. A contact profilometry instrument (KLA-Tencor, Milipitas, CA, USA) was used to measure and control the Ru-W film thickness.

### 3.2. Cu Electroplating

For the electrodeposition of Cu on Ru-W/SiO_2_/Si substrates, an acidic electrolyte was used, containing 0.05 M CuSO_4_∙5H_2_O (99.995%, Sigma-Aldrich, St. Louis, MO, USA), 0.05 M H_2_SO_4_ (Honeywell/Fluka, Charlotte, NC, USA), 1 mM NaCl (Honeywell/Fluka, Charlotte, NC, USA), and 300 ppm poly(ethylene glycol) 600 (Fluka Chemie GmbH, Buchs, Switzerland) in deionized water. A two-electrode configuration was employed with the Ru-W/SiO_2_/Si substrates working as the cathode and a copper plate as the anode placed at a fixed distance of 65 mm from the cathode. 

Electrodepositions were performed at room temperature and without electrolyte stirring using a Gamry potentiostat/galvanostat Interface 1000E (Gamry Instruments, Warminster, PA, USA). Additional details on the materials and electrolyte pH measurement can be found in [12]. The substrates were covered with a polymeric mask, limiting the exposed area to a circle of 0.50 cm^2^. After electroplating, the substrates were rinsed in deionised water and dried with a gentle Ar blow.

### 3.3. Structural and Chemical Characterisation of Substrate and Cu Films

The sputtered PVD Ru-W/SiO_2_/Si substrate surface was observed by scanning electron microscopy (SEM) (Thermo Fisher Scientific Quanta 400/650FEG ESEM, Thermo Fisher Scientific, Hillsboro, OR, USA) and by atomic force microscopy (AFM) (Veeco Metrology Multimode, Veeco Instruments Inc., Oyster Bay, NY, USA) with a Bruker TESPA-V2 tip. The chemical composition of the Ru-W thin films was estimated by energy-dispersive X-ray spectroscopy (EDS) (EDAX Genesis X4M, AMETEK, Berwyn, PA, USA), while its structure was determined by grazing incidence X-ray diffraction (GIXRD) (Bruker D8 Discover, Bruker Corporation, Billerica, MA, USA) at an angle of 1.5° using Cu Kα radiation (λ = 1.54184 Å) and a step of 0.04°·s^−1^. 

The electrodeposits of Cu/Ru-W films were analysed by SEM (Thermo Fisher Scientific Quanta 650FEG ESEM, Thermo Fisher Scientific, Hillsboro, OR, USA) and digital microscopy (Leica DVM6, Leica Microsystems GmbH, Wetzlar, Germany). The Cu-substrate interface cross-section was prepared by a focused ion-beam (FIB) (Thermo Fisher Scientific Helios 450S, Thermo Fisher Scientific, Hillsboro, OR, USA) and observed by scanning transmission electron microscopy (TEM) (Thermo Fisher Scientific Titan G2 ChemiSTEM) with EDS mapping. Image analyses were performed on the *ImageJ* software version 1.51p (National Institute of Health, Bethesda, MD, USA). 

## 4. Conclusions

Cu was electroplated directly on Ru-W thin films with near equimolar composition to create candidates to replace the conventional Ta/TaN as seedless diffusion barrier layers for advanced Cu interconnect metallisation. Direct and pulse-reverse current depositions were galvanostatically applied in a conventional acidic Cu electrolyte with additives. The main conclusions of this study are:

▪ Thin Cu films can be directly electroplated on near equimolar Ru-W thin films using a conventional acidic electrolyte.
▪ Under the flat substrate configuration and electroplating conditions herein utilised, a minimum average current density between 3 and 5 mA·cm^−2^ was required to achieve substrate coverage, regardless of the current application mode.
▪ A wide range of particle sizes formed on the substrate. A variable thickness, compact Cu film was partially interrupted by large star-like/dendritic Cu particles that prevented substrate coverage in their vicinity.
▪ A pulse-reverse current effectively reduced the particle size, which contributed to a smoother film but appeared unable to eliminate large Cu particles. Hydrogen evolution was noticeable above 6 mA·cm^−2^.
▪ Substrate coverage was not complete, and this requires improvement; however, a continuous interface between Cu and Ru-W was formed.

## Figures and Tables

**Table 1:** The direct (DC) and pulse-reverse current (PRC) Cu electroplating conditions utilised.

Condition	J mA·cm^−2^	t s	Q mC	$j_{c}$ mA·cm^−2^	$j_{a}$ mA·cm^−2^	$t_{c}$ ms	$t_{a}$ ms
DC-2	2	150	300	-	-	-	-
DC-5	5	60	300	-	-	-	-
DC-10	10	30	300	-	-	-	-
PRC-5	3	100	300	5	5	20	5
PRC-10	6	50	300	10	10	20	5
PRC-20	12	25	300	20	20	20	5

## Data Availability

The data presented in this study are available on request from the corresponding author.
